# Derotational osteotomy is a relevant procedure in the management of lateral patellar dislocation: An expert survey of the International Patellofemoral Study Group

**DOI:** 10.1002/jeo2.70116

**Published:** 2025-02-12

**Authors:** Guido Wierer, Danko Milinkovic, Gerd Seitlinger, Michael Liebensteiner, William Post, Philipp W. Winkler

**Affiliations:** ^1^ Department of Orthopedics and Traumatology Paracelsus Medical University Salzburg Austria; ^2^ Research Unit for Orthopaedic Sports Medicine and Injury Prevention Private University for Health Sciences, Medical Informatics and Technology Hall in Tirol Austria; ^3^ Center for Musculoskeletal Surgery Charité‐University Medicine Berlin Berlin Germany; ^4^ Orthofocus Salzburg Austria; ^5^ Orthopädie für Hüfte, Knie & Fuß im Zentrum Innsbruck Austria; ^6^ Private Hospital Kettenbrücke Innsbruck Austria; ^7^ Mountaineer Orthopedic Specialists Morgantown West Virginia USA; ^8^ Department for Orthopaedics and Traumatology Kepler University Hospital GmbH, Johannes Kepler University Linz Linz Austria

**Keywords:** alignment, instability, osteotomy, patella, rotation, survey, torsion

## Abstract

**Purpose:**

To evaluate current knowledge and discover potential controversies in treating torsional deformities of the lower limb in patients with lateral patellar dislocation (LPD) among patellofemoral experts.

**Methods:**

An online survey was distributed to all active International Patellofemoral Study Group (IPSG) members, representing an international sample of orthopaedic surgeons with a specific interest and experience in patellofemoral joint disorders. The survey included 21 single‐ and multiple‐choice questions and was distributed by email between 2022 and 2023.

**Results:**

Thirty‐five members (54%) completed the questionnaire. The responding experts conduct a hip–knee–ankle magnetic resonance imaging or computed tomography following first‐time and recurrent patellar dislocation based on clinical examination (43% and 49%, respectively), routinely (6% and 23%, respectively), or not at all (51% and 29%, respectively). Two thirds of the experts perform derotational osteotomies of the femur, and 37% perform derotational osteotomies of the tibia. Most of these surgeons (61% and 69%, respectively) perform less than five derotational osteotomies of the femur or tibia per year. The most important factors for performing derotational osteotomy are abnormal torsion (100%), abnormal gait pattern (57%), revision cases (74%) and recurrent patellar instability (61%). Most surgeons (65%) agree on a cut‐off value of >30° of femoral ante‐torsion and >35° of external tibial torsion to perform derotational osteotomy, but the preferred measurement techniques vary.

**Conclusion:**

Torsional deformities of the lower limb are a clinically relevant topic in the management of patients with recurrent LPD. Although the caseload is low, most experts perform derotational osteotomies. Diagnostic and therapeutic algorithms overlap widely between surgeons, but the indication and cut‐off values for performing derotational osteotomy must be further established.

**Study Design:**

Survey.

**Level of Evidence:**

Level V.

**Abbreviations**
CT: computed tomography
IPSG: International Patellofemoral Study Group
LPD: lateral patellar dislocation
MRI: magnetic resonance imaging

## INTRODUCTION

Recurrent lateral patellar dislocation (LPD) is a multifactorial condition occurring in 30%–57% of patients after a first‐time LPD [4, 27]. Recurrent LPD leads to considerable impairment in the quality of life of affected patients and is associated with modifiable and non‐modifiable risk factors [1, 4, 5, 6, 19, 27]. Underlying risk factors should be analyzed in all patients with LPD and, if necessary, addressed to improve patient outcomes [20]. Modifiable risk factors for recurrent LPD have been reported in numerous studies and include insufficiency of the medial soft tissue restraints, trochlear dysplasia, patellar maltracking and lower limb deformity [3, 6, 15, 27].

Coronal (i.e., valgus deformity) and axial (i.e., torsional deformity) lower limb deformities have long been underestimated in treating recurrent LPD. Valgus deformity of the lower limb has become a recognized risk factor for recurrent LPD and is now regularly treated by corrective osteotomies [9, 10]. Despite a limited body of evidence, torsional deformities of the femur (i.e., excessive femoral ante‐torsion) and the tibia (i.e., excessive external torsion of the tibia) are receiving increasing attention in the management of recurrent LPD [2, 8, 23, 26, 28]. Nevertheless, there is still a lack of consensus on diagnostic workup, indications, and surgical techniques for the treatment of torsional deformities.

Derotational osteotomies of the femur and the tibia have been associated with favourable clinical outcomes and a low rate of recurrent LPD [2, 8, 22, 23, 26, 30]. Given the clinical complexity of torsional deformities and the overall limited evidence, the indication for derotational osteotomies is still controversial. In addition, several techniques to measure femoral and tibial torsion with a wide range of reference values have been reported [13, 17, 18]. The limited consensus on torsional measurement techniques and the indication for derotational osteotomies warrant further investigation. The International Patellofemoral Study Group (IPSG) consists of international experts in the management of patellofemoral joint disorders. With many years of clinical experience and ongoing scientific activity, the members of the IPSG endeavour to bring consensus to controversial topics in patellofemoral joint disorders [20]. Expert opinions are valuable in clinical decision‐making on controversial issues like managing torsional deformities.

The purpose of this study was to evaluate current knowledge and discover potential controversies among IPSG members regarding the treatment of torsional deformities of the lower limb in patients with LPD.

## MATERIALS AND METHODS

An online survey (LimeSurvey GmbH, version 5.0) was distributed to all active IPSG members, representing an international sample of orthopaedic surgeons with a specific interest and experience in patellofemoral joint disorders. The survey included 21 single‐ and multiple‐choice questions (Appendix S1) that were formulated by the ‘question group’ (G.S., M.L. and W.P.) and reviewed by the ‘literature group’ (G.W., D.M. and P.W.) based on a systematic review of the literature. The survey was distributed by email between November 2022 and July 2023. The first part of the questionnaire investigated the participants' demographics, diagnostic workup after patellar dislocation and preferred measuring technique for femoral and tibial torsion.

In the second and third parts of the survey, participants performing derotational osteotomies of the femur and/or tibia were further questioned. The case load, preferred surgical procedure and relevant factors for performing derotational osteotomies were investigated. In addition, the surgeons' cut‐off and target values were collected according to their preferred femoral and tibial measurement techniques.

### Statistical analysis

The study's results were presented using descriptive statistics and bar charts. The torsional cut‐off and target values were reported as mean and 95% confidence interval using forest plots. Excel (Microsoft, 2023) was used for data analysis.

## RESULTS

**Figure 1:**
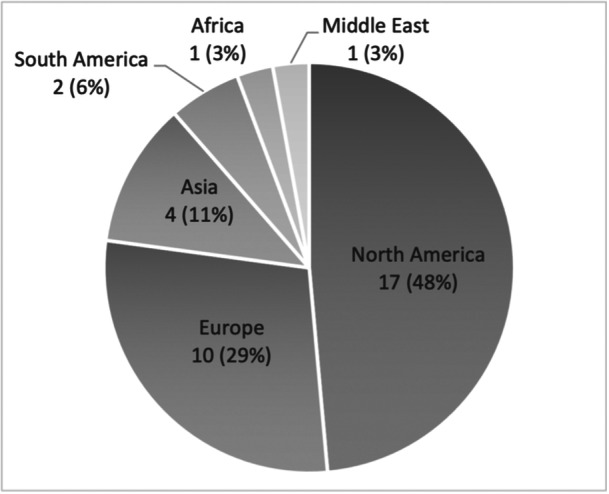
The diagram illustrates the geographical distribution of the survey's participating surgeons in absolute and relative (in brackets) numbers.

Figure 1 shows the geographical distribution of the 35 IPSG members (54%) who completed the questionnaire. Most members, 22 out of 35 (63%), have been treating patellofemoral disorders for more than 15 years. Another four (11%) and nine (26%) have been in practice for 5–10 years and 11–15 years, respectively.

**Figure 2:**
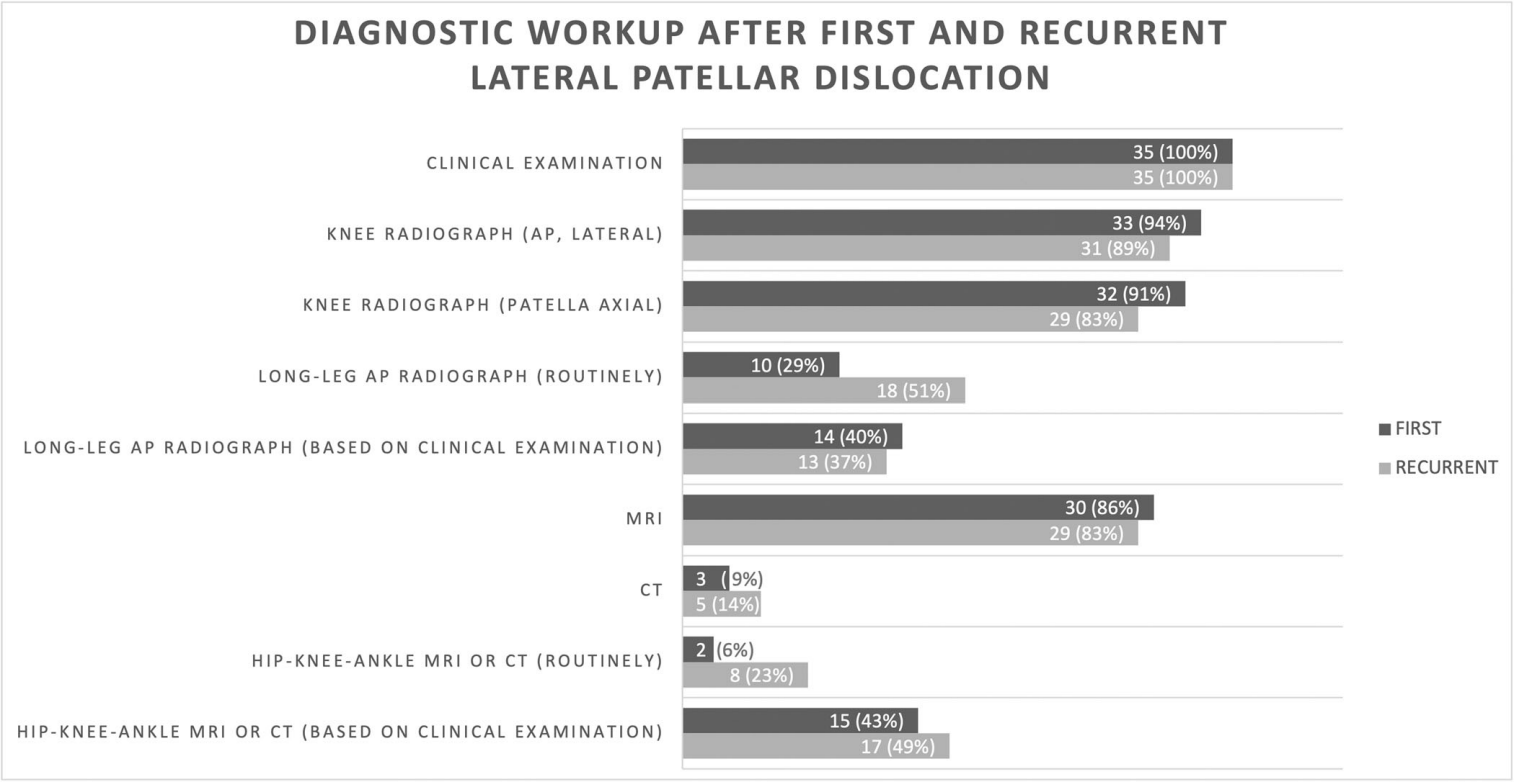
The diagram shows the absolute numbers and percentage of surgeons performing a diagnostic examination after a first or recurrent lateral patellar dislocation.

The experts conduct hip–knee–ankle magnetic resonance imaging (MRI) or computed tomography (CT) scans following first and recurrent LPD based on clinical examination (43% [*n* = 15] and 49% [*n* = 17], respectively), routinely (6% [*n* = 2] and 23% [*n* = 8], respectively) or not at all (51% [*n* = 18] and 29% [*n* = 10], respectively). The detailed results regarding the diagnostic workup after first‐time and recurrent LPD are shown in Figure 2.

### Femoral deformities

The most preferred technique for measuring femoral torsion, according to 54% of the participants (*n* = 19), is the angle between a line parallel to the femoral neck and a line parallel to the posterior femoral condyles (e.g., Jarrett et al. [16]). The second most common technique (31%, *n* = 11) is a line parallel to the posterior femoral condyles and a line connecting the centre of the femoral head and the centre of the greater trochanter (e.g., Waidelich et al. [25]). Additional quoted techniques included Murphy's (6%, *n* = 2) [21] and Yoshioka's (3%, *n* = 1) [29]. One surgeon (3%) prefers clinical measurement.

**Figure 3:**
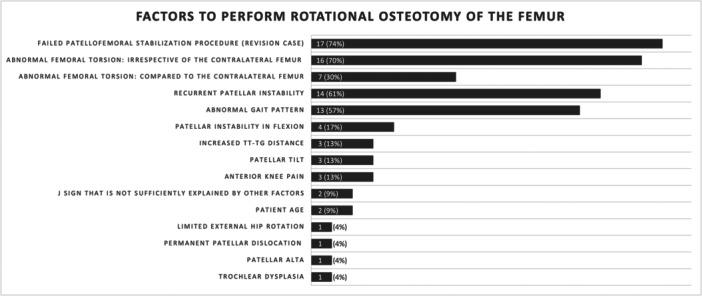
The diagram shows the absolute numbers and percentage of surgeons considering a derotational osteotomy of the femur according to the respective factor.

Two thirds of the experts (*n* = 23) perform derotational osteotomies of the femur in patients with patellofemoral instability, and most (61%, *n* = 14) perform less than five per year. Only two surgeons (9%) perform more than 10 per year. The criteria for performing derotational osteotomy of the femur are mentioned in Figure 3, including revision cases, abnormal femoral torsion, recurrent patellar instability, an abnormal gait pattern and patellar instability in flexion.

**Figure 4:**
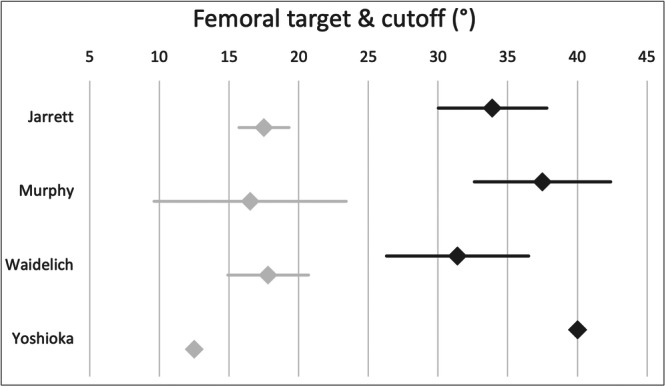
The diagram shows the surgeons' cut‐off values (mean, 95% confidence interval) for performing derotational osteotomy of the femur (dark grey) and the targeted values of femoral ante‐torsion post‐surgery (light grey) according to their preferred measuring technique.

Figure 4 shows the cut‐off values for performing derotational osteotomy of the femur. The majority of participants (65%, *n* = 16) agree on a cut‐off value greater than 30°. Another 17% (*n* = 4) refer to clinical signs only, including gait analysis and an inwardly pointing knee, and 30% of the surgeons (*n* = 7) consider the contralateral side.

The preferred techniques to perform derotational osteotomy of the femur are mono‐ and bi‐planar supracondylar osteotomy (35% *[n* = 8] and 30% [*n* = 7], respectively), followed by inter‐trochanteric and sub‐trochanteric/midshaft osteotomy (9% [*n* = 2] and 9% [*n* = 2], respectively). Seventeen per cent of the surgeons (*n* = 4) prefer to consider the deformity's location when choosing the osteotomy site, although this would imply measuring femoral torsion at several sections separately.

Most participants (87%, *n* = 20) aim for a femoral ante‐torsion between 15° and 20°. Figure 4 shows the results of the targeted values of femoral torsion post‐surgery.

**Figure 5:**
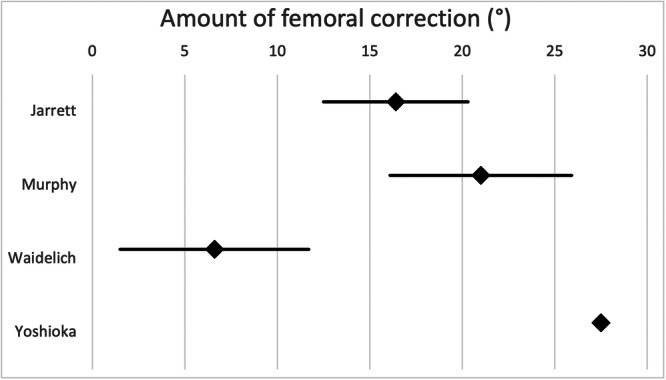
The diagram shows the amount of femoral derotation (mean, 95% confidence interval) the surgeons aim for according to their preferred measurement technique.

The mean difference between the femoral cut‐off and target value, or the average amount of femoral derotation, varies between surgeons according to their preferred torsion measurement technique, as shown in Figure 5.

### Tibial deformities

The most preferred technique for measuring tibial torsion is the angle between a line parallel to the posterior tibial condyles and a line connecting the centre of the medial and lateral malleolus (e.g., Goutallier et al. [11]), according to 63% of the participants (*n* = 22). The second most common technique (31%, *n* = 11) is the angle between a line parallel to posterior tibial condyles and a line connecting the centre of the pilon tibiale and the centre of the fibular incision (e.g., Waidelich et al. [25]). Another quoted measurement was the angle between a line parallel to the posterior tibial condyles and the anterior talus (3%, *n* = 1). One surgeon (3%) prefers clinical measurement.

**Figure 6:**
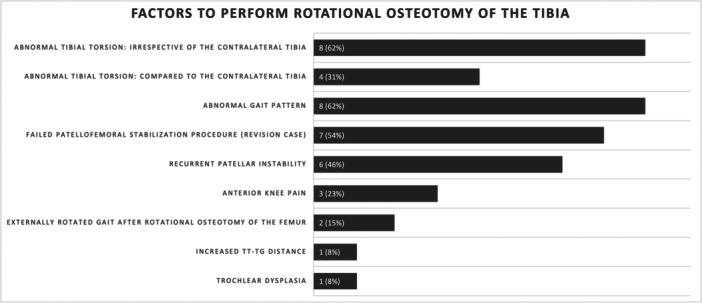
The diagram shows the absolute numbers and percentage of surgeons considering a derotational osteotomy of the tibia according to the respective factor.

Thirteen out of 35 experts (37%) perform derotational osteotomies of the tibia in patients with patellofemoral instability, and most of these surgeons (69%, *n* = 9) perform less than five per year. Figure 6 mentions the criteria for performing derotational osteotomies of the tibia, including abnormal tibial torsion, abnormal gait pattern, revision cases and recurrent patellar instability.

**Figure 7:**
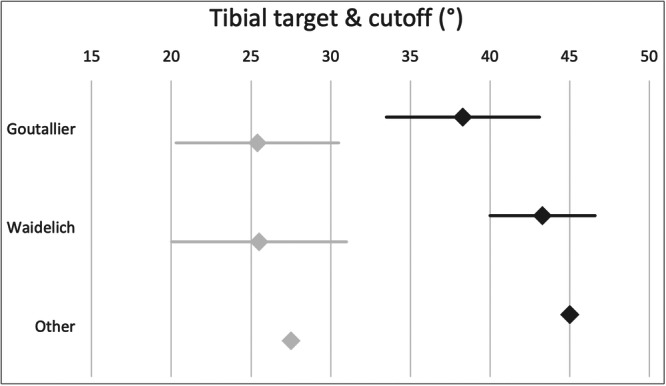
The diagram shows the surgeons' cut‐off values (mean, 95% confidence interval) for performing derotational osteotomy of the tibia (dark grey) and the targeted values of tibial external torsion post‐surgery (light grey) according to their preferred measuring technique.

Figure 7 shows the cut‐off values for performing internal rotational osteotomy of the tibia. Most participants (69%, *n* = 9) agree on a cut‐off value greater than 35°. Another 23% (*n* = 3) refer to clinical signs only, and 15% of the surgeons (*n* = 2) consider the contralateral side.

The preferred techniques to perform derotational osteotomy of the tibia are supra‐ and infratuberositary osteotomy (23% [*n* = 3] and 23% [n = 3], respectively), followed by midshaft osteotomy (15%, *n* = 2) and transtuberositary osteotomy (15% [*n* = 2] mono‐planar with detachment of the tibial tuberosity, and 8% [*n* = 1] bi‐planar ascending). One surgeon (8%) prefers supramalleolar osteotomy, and another surgeon (8%) prefers to consider the deformity's location when choosing the osteotomy site.

Most participants (77%, *n* = 10) aim for a value between 20° and 30° of tibial external torsion. Figure 7 shows the results of the targeted range of tibial torsion post‐surgery.

**Figure 8:**
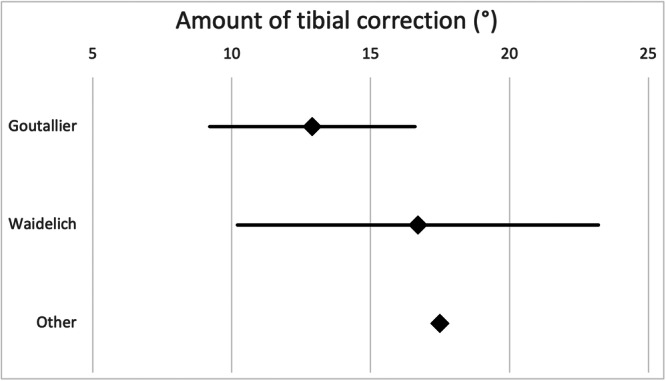
The diagram shows the amount of tibial derotation (mean, 95% confidence interval) the surgeons aim for according to their preferred measurement technique.

Figure 8 shows the average amount of tibial derotation performed according to the surgeons' preferred measurement technique.

## DISCUSSION

The most important finding of this study was that global experts consider torsional deformity of the lower limb to be a clinically relevant topic in the management of patients with recurrent LPD. Although the caseload is low, most experts perform derotational osteotomies of the femur and the tibia. However, there is still disagreement about the diagnostic workup in patients with torsional deformity and the indication for derotational osteotomies.

Recent findings have confirmed that there still needs to be a consensus on the ideal measurement technique to determine the torsion of the femur and the tibia [2]. Various techniques to determine femoral torsion have been published, which differ mainly in determining the femoral neck axis [2, 17]. One study found significant differences in femoral ante‐torsion between six different measurement techniques using CT scans [17]. The highest mean values for femoral ante‐torsion were observed with the Waidelich technique, which differed by up to 16° from other measurement techniques [17]. Such significant differences indicate an urgent need for a consensus to determine femoral torsion. Unfortunately, no comparable studies evaluate different measurement techniques for tibial torsion.

This study showed that experts worldwide use the technique described by Jarett et al. (54%) and Waidelich et al. (31%) to determine femoral torsion. Tibial torsion is most commonly defined according to Goutallier et al. (63%) and Waidelich et al. (31%) by study participants. Good inter‐ and intra‐rater reliability has been shown for different measurement techniques [17]. Therefore, which method is used may be irrelevant. More importantly, the indication for derotational osteotomy needs to be established for a single measurement technique and imaging modality. Although CT scans are considered the gold standard for determining lower limb torsion [2], MRI scans are widely used according to the present study and are an equivalent alternative [12].

In this cohort of highly experienced experts in treating LPD, clinical examination, knee radiography, and MRI represent the cornerstones of diagnostics after first‐time and recurrent LPD. Hip–knee–ankle MRI/CT scans are mainly acquired based on clinical examination. Surprisingly, 51% and 28% of the experts do not perform hip‐knee‐ankle MRI/CT scans after first and recurrent LPD, respectively. Considering the available evidence that lower limb torsional malalignment is a risk factor for recurrent LPD [2, 8, 23, 26, 28], these numbers are an interesting finding.

Derotational osteotomy of the femur has been proven to be an effective complementary procedure in treating patellofemoral instability in patients with excessive femoral ante‐torsion [23]. A recent systematic review, including ten studies, evaluated patients' clinical and functional outcomes with patellofemoral instability undergoing derotational distal femoral osteotomy. Overall, 309 knees (295 patients) with excessive femoral ante‐torsion were included [23]. After a weighted average follow‐up of more than three years and a pooled mean femoral derotation of 19.4°, significant clinical improvements were observed, consistently exceeding the minimal clinically important difference [23]. The clinical effectiveness of derotational femoral osteotomy was underscored by an overall satisfaction rate of 93% [23]. Comparable results were confirmed by other investigations [2, 7, 14, 26]. Two‐thirds of the experts in this study perform derotational femoral osteotomies. However, 61% of the experts perform less than five derotational osteotomies per year. Based on a recent systematic review, the cut‐off to perform derotational osteotomy of the femur is >25° of femoral ante‐torsion [2]. This is similar to the results of this study, in which 65% of participants agreed with a cut‐off value of >30° to perform derotational osteotomy of the femur irrespective of the applied measurement technique.

**Figure 9:**
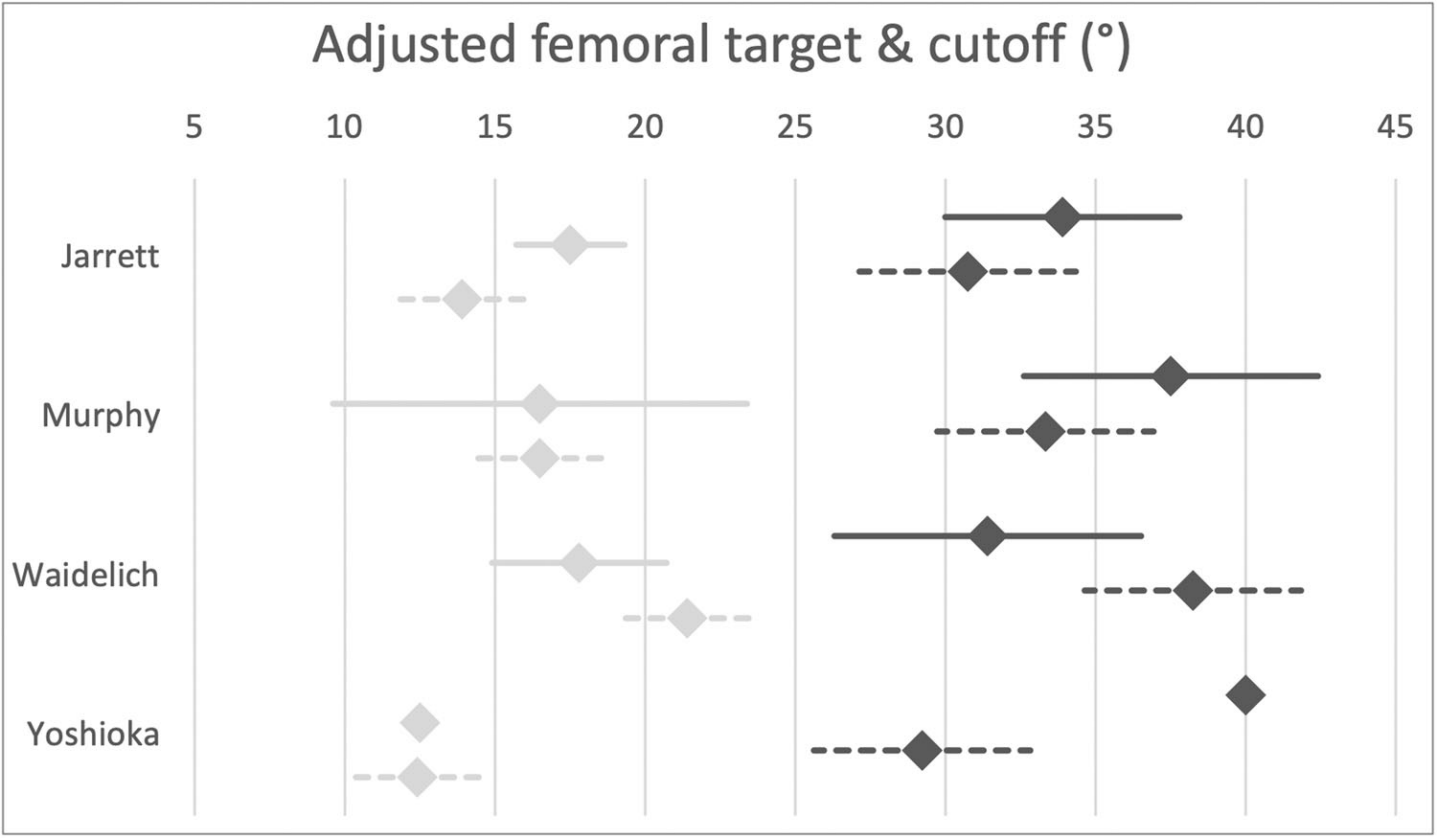
The diagram shows the surgeons' cut‐off values (mean, 95% confidence interval) for performing derotational osteotomy of the femur (dark grey) and the targeted values of femoral ante‐torsion post‐surgery (light grey) according to their preferred measuring technique. The reported values with respect to the preferred technique were adjusted (dashed line) to all cited measurement techniques according to the reported differences by Kaiser et al. [17].

However, it is crucial to consider the differences between the various measurement techniques when stating a specific cut‐off value. Kaiser et al. [17] reported a mean femoral ante‐torsion of 22.4°, 17.5°, 14.9° and 13.4° when applying Waidelich's, Murphy's, Jarrett's and Yoshioka's measurement technique in the same patient cohort, respectively. Considering these differences, the stated cut‐off and target values in the present survey were adjusted from the surgeons' preferred measuring technique to all cited techniques, as shown in Figure 9. Interestingly, those surgeons who use Waidelich's technique quoted a lower cut‐off value (31.4°) than the surgeons who use Murphy's (37.5°) or Jarrett's (33.9°) technique, which was found to be 4.9° and 7.5° lower than Waidelich's technique, respectively [17]. According to Kaiser et al. [17], surgeons using Waidelich's technique should aim for higher cut‐off values than those using Murphy's or Jarrett's technique.

Of note, this study showed that 70% of experts consider abnormal femoral torsion a major indication of derotational osteotomy. Revision cases (74%), recurrent LPD (61%), abnormal gait pattern (57%) and patellofemoral instability in flexion (17%) represent more major criteria for considering derotational osteotomy of the femur. This aligns with a systematic review reporting that clinical examination, CT and MRI are used in studies evaluating torsional malalignment [2].

In this group of experts, 37% perform derotational osteotomies of the tibia, with 69% of experts performing less than five per year. Based on a recent systematic review, the cut‐off to perform derotational osteotomy of the tibia is >30° of external tibial torsion [2]. In this study, 69% of participants agreed on a cut‐off value of >35° to perform derotational osteotomy of the tibia. Interestingly, 23% of experts refer to clinical signs only, and 15% consider the contralateral tibia as an indication of osteotomy. It is important to remember that the degree of torsion in the femur and tibia must be considered together, and it is likely best to combine them with other variables, such as soft tissue laxity and bony deformity of the trochlea, to determine the optimal treatment strategy [24].

Although favourable results after derotational osteotomy have been published, osteotomy‐related complications such as knee stiffness, persistent knee pain, painful hardware, thrombosis, non‐union of the osteotomy gap, peroneal nerve irritation and loss of correction have been reported and need to be considered in treatment decision‐making [2, 8, 23, 26]. In addition, the limited quality of evidence with a high risk of bias and heterogeneous patient cohorts should be considered when interpreting such study results.

Some limitations of this study should be acknowledged. This study surveyed international experts in the management of patellofemoral joint disorders and represents an expert opinion rather than a consensus statement. However, disagreements in the management of torsional deformities in patients with recurrent LPD were identified, which may form the basis for future study as we work toward consensus. Another limitation is the rather low response rate to the questionnaire and the resulting low number of participants.

## CONCLUSION

Torsional deformities of the lower limb are a clinically relevant topic in the management of patients with recurrent LPD. Although the caseload is low, most experts perform derotational osteotomies. Diagnostic and therapeutic algorithms overlap widely between surgeons, but the indication and cut‐off values for performing derotational osteotomy must be established.

## AUTHOR CONTRIBUTIONS

All listed authors have contributed substantially to this work. Guido Wierer, Danko Milinkovic and Philipp W. Winkler performed the literature review and primary manuscript preparation. Guido Wierer, Philipp W. Winkler, Gerd Seitlinger, Michael Liebensteiner and William Post established the survey and assisted with the interpretation of the results, initial drafting of the manuscript, as well as editing and final manuscript preparation. All authors read and approved the final manuscript.

## CONFLICT OF INTEREST STATEMENT

The authors declare no conflicts of interest.

## ETHICS STATEMENT

No ethical approval was required for this survey. Informed consent was obtained from all participants included in the study.

## Supporting information

Supporting information.

## Data Availability

The analyzed data reported in this study are available on request from the corresponding author.
