# Supplementary material for: Derotational osteotomy is a relevant procedure in the management of lateral patellar dislocation: An expert survey of the International Patellofemoral Study Group
Source: J Exp Orthop. 2025 Feb 12;12(1):e70116. doi: 10.1002/jeo2.70116 (PMC11821726; doi:10.1002/jeo2.70116)
Supplement: Supplementary file 1 — Supporting information. [file JEO2-12-e70116-s001.pdf]

Derotational osteotomy is a relevant procedure in the management of lateral patellar dislocation:
An Expert Survey of the International Patellofemoral Study Group.

1. Where do you practice?
   (Single Choice)

   a. Europe
   b. Asia
   c. US
   d. North America (outside US)
   e. South America
   f. Australia
   g. Other

2. How many years have you been in practice in the treatment of patellofemoral disorders?
   (Single Choice)

   a. <5 years
   b. 5-10 years
   c. 11-15 years
   d. >15 years

3. How many patellofemoral instability cases do you treat annually?
   (Single Choice)

   a. <25
   b. 25-50
   c. 50-100
   d. >100

4. What is your diagnostic workup after first-time patellar dislocation?
   (Multiple Choice)

   a. Clinical examination
   b. Knee radiograph: anterior-posterior & lateral
   c. Knee radiograph: axial
   d. Long-leg ap radiograph: routinely
   e. Long-leg ap radiograph: based on clinical examination
   f. MRI
   g. CT
   h. Hip-Knee-Ankle MRI or CT: routinely
   i. Hip-Knee-Ankle MRI or CT: based on clinical examination

5. What is your diagnostic workup after recurrent patellar dislocation?
   (Multiple Choice)

   a. Clinical examination
   b. Knee radiograph: anterior-posterior & lateral
   c. Knee radiograph: axial
   d. Long-leg ap radiograph: routinely
   e. Long-leg ap radiograph: based on clinical examination
   f. MRI
   g. CT
   h. Hip-Knee-Ankle MRI or CT: routinely
   i. Hip-Knee-Ankle MRI or CT: based on clinical examination

6. What is your preferred technique for measuring femoral torsion?
   (Single Choice)

a. Angle between a line parallel to posterior femoral condyles and a line connecting the center of the femoral head and the center of the greater trochanter (e.g. Waidelich et al.)
b. Angle between a line parallel to posterior femoral condyles and a line parallel to the femoral neck (e.g. Jarrett et al.)
c. Other (please specify)

7. What is your preferred technique for measuring tibial torsion?
(Single Choice)

    a. Angle between a line parallel to posterior tibial condyles and a line connecting the center of the pilon tibiale and the center of the fibular incision (e.g. *Waidelich et al.*)
    b. Angle between a line parallel to posterior tibial condyles and a line connecting the center of the medial and lateral malleolus (e.g. *Goutallier et al.*)
    c. Other (please specify)

8. Do you perform lower limb osteotomies?
(Single Choice)

    a. Yes
    b. No

9. How many lower limb osteotomies do you perform annually?
(Single Choice)

    a. <5
    b. 6-10
    c. 11-20
    d. 21-30
    e. >30

---

10. Do you perform rotational osteotomies of the femur in patients with patellofemoral instability?
(Single Choice)

    a. Yes
    b. No, but I refer patients for rotational. osteotomies
    c. No

11. How many rotational osteotomies of the femur in patients with patellofemoral instability do you perform annually?
(Single Choice)

    a. <5
    b. 6-10
    c. 11-20
    d. 21-30
    e. >30

12. What is your cutoff value of femoral ante-torsion to perform torsional osteotomy of the femur?
(Multiple choice)
    a. 15°
    b. 20°
    c. 25°
    d. 30°
    e. 35°
    f. 40°
    g. Depending on the contralateral torsion

h. Depending on clinical signs (gait analysis, inwardly pointing knee etc.)

13. What is your preferred technique to perform rotational osteotomy of the femur?
(Single Choice)

   a. Supracondylar mono-planar
   b. Supracondylar bi-planar
   c. Subtrochanteric
   d. Depending on the location of the deformity
   e. Other (please specify)

14. What is your target range for femoral torsion?
(Free text)

15. What are the most important factors for you to perform rotational osteotomy of the femur?
(Multiple choice)

   a. Abnormal torsion of the index femur: *irrespective* of the contralateral femur
   b. Abnormal torsion of the index femur: *compared* to the contralateral femur
   c. Recurrent patellar instability
   d. Failed patellofemoral stabilization procedure (i.e., revision case)
   e. Increased TT-TG distance
   f. Patellar alta
   g. Trochlea dysplasia
   h. Patellar instability in flexion
   i. Patellar tilt
   j. Patient age
   k. Abnormal gait pattern
   l. Other (please specify)

---

16. Do you perform rotational osteotomies of the tibia in patients with patellofemoral instability?
(Single Choice)
   a. Yes
   b. No, but I refer patients for rotational. osteotomies
   c. No

17. How many rotational osteotomies of the tibia in patients with patellofemoral instability do you perform annually?
(Single Choice)
   a. <5
   b. 6-10
   c. 11-20
   d. 21-30
   e. >30

18. What is your cutoff value of external tibial torsion to perform rotational osteotomy of the tibia?
(Multiple choice)
   a. 25°
   b. 30°
   c. 35°
   d. 40°
   e. 45
   f. Depending on the contralateral torsion
   g. Depending on clinical signs (gait analysis, inwardly pointing knee etc.)

19. What is your preferred technique to perform rotational osteotomy of the tibia?
(Single Choice)
   a. Supratuberositary

b. Infratuberositary
c. Transtuberositary – Biplanar ascending
d. Transtuberositary – Biplanar descending
e. Transtuberositary – Monoplanar with detachment of the tibial tuberosity
f. Supramalleolar
g. Depending on the location. of the deformity
h. Other (please specify)

20. What is your target range for tibial torsion?
(Free text)

21. What are the most important factors for you to perform rotational osteotomy of the tibia?
(Multiple choice)

a. Abnormal torsion of the index femur: *irrespective* of the contralateral femur
b. Abnormal torsion of the index femur: *compared* to the contralateral femur
c. Recurrent patellar instability
d. Failed patellofemoral stabilization procedure (i.e., revision case)
e. Increased TT-TG distance
f. Patellar alta
g. Trochlea dysplasia
h. Patellar instability in flexion
i. Patellar tilt
j. Patient age
k. Abnormal gait pattern
l. Other (please specify)
